# Study on the Effect of Corrosion on Tensile Properties of GW63K Magnesium Alloy

**DOI:** 10.3390/ma18102244

**Published:** 2025-05-12

**Authors:** Tian Lan, Feng Ye, Binbin Liu, Haoyang Du

**Affiliations:** 1 State Key Laboratory for Advanced Metals and Materials, University of Science and Technology Beijing, 30 Xueyuan Road, Beijing 100083, China; lantian11007@foxmail.com (T.L.); bbliu@ustb.edu.cn (B.L.); 2 State Grid Jilin Electric Power Research Institute, No. 4433 Renmin Street, Changchun 130021, China

**Keywords:** corrosion, mechanical property, magnesium alloy, corrosion residual strength, rare earth element

## Abstract

Tensile tests of GW63K (Mg-6Gd-3Y-Zr) magnesium alloy under different heat treatment conditions were performed after immersion of the test specimens in 3.5 wt.% NaCl aqueous solution for periods up to 72 h. Results indicate that pitting corrosion should be responsible for the severe degradation of the tensile properties including yield strength, tensile strength (corrosion residual strength), and elongation within the testing time. The extreme depth of the corrosion pit was statistically related to corrosion residual strength. The variation of corrosion residual strength exhibited an exponential decay trend from 0 to 72 h. Furthermore, heat treatment enhanced the corrosion resistance of the GW63K alloy by inhibiting both the initiation and growth of corrosion pits, thereby improving the corrosion residual strength of the alloy. The critical working strength of GW63K alloy was significantly improved (increased to 192 MPa from 132 MPa) via heat treatment.

## 1. Introduction

The demand for high-strength, lightweight structural materials in navigation, aviation, and aerospace applications has grown significantly in recent years. Magnesium alloys have attracted considerable attention in the above-mentioned area due to their low density and high specific strength [1,2]. However, the inherent chemical reactivity and limited mechanical properties at ambient temperature of magnesium alloys have restricted their widespread adoption [3,4].

Alloying with rare earth (RE) elements has proven to be an effective way to enhance the performance of Mg alloys. Many researchers have reported that RE-Mg alloys exhibit superior tensile strength at both room and elevated temperatures, along with improved corrosion resistance in comparison to conventional commercial Mg alloys [5,6,7,8,9,10,11,12,13]. For instance, artificially aged GW63K (Mg-6Gd-3Y-Zr, wt.%) Mg alloy achieves a room-temperature tensile strength exceeding 300 MPa, while the tensile strength of AZ91 alloy, which is one of the most widely used traditional commercial Mg alloys, is 250 MPa on average. It is reported that GW63K alloy can achieve certain levels of strength and corrosion resistance via heat treatment that the performance is vastly improved [7,8,9,14]. The as-cast GW63K alloy contains compounds comprising Gd and Y elements. Following solution treatment, these eutectic compounds dissolve, with Gd and Y atoms entering the matrix and inducing lattice distortion, thereby enhancing both strength and elongation of the alloy. Subsequent aging treatment leads to the precipitation of secondary phases, which further improves the alloy’s strength while marginally compromising its ductility. Therefore, GW63K possesses great potential for widespread application in the high-tech industrial sectors as mentioned above.

However, due to the reactive chemical properties of the Mg element, magnesium alloys are usually susceptible to corrosion that will deteriorate the mechanical properties. The studies have shown the connection between corrosion and the reduction of mechanical properties of GW63K alloy [6,7]. In general, pitting corrosion is the main factor leading to a decrease in the strength of Mg alloys in corrosive environments [15,16,17]. Corrosion pits can cause stress concentrations at the bottom of the pits and a decrease in the cross-section of corroded metals or alloys. As a result, the cracks are induced under lower stress conditions than expected. The phenomenon is known as stress corrosion cracking (SCC), which is extremely dangerous and complicated in real industries since it usually leads to sudden fracture and then to catastrophic accidents [3,18]. The dimensions of the depth of the corrosion pits play a great role in the AZ91 alloy. Wang et al. [15,16] revealed that in AZ91 alloy with 1.0 wt.% Y, the variation of the corrosion residual strength (CRS) is linearly dependent on the extreme depth of the corrosion pits (EDCP). The result was also reported by Li et al. [17] in AZ91 alloy with 1.0 wt.% Ce. Corrosion may also attack the fatigue behavior of Mg alloys. For instance, the fatigue strength of Mg-7%Gd-5%Y-1%Nd-0.5%Zr alloy is decreased for 25% in NaCl aqueous solution [19]. Hydrogen embrittlement and crack initiation at multiple sites of grains extremely shorten the fatigue life of alloy.

Although a number of papers revealed varied corrosion performance of GW63K alloy [6,7], the effect of corrosion on mechanical properties of GW63K alloy is still ambiguous and needs further investigation. The present study aims to investigate the effect of corrosion on the tensile property of GW63K (Mg-6Gd-3Y-Zr) magnesium alloys in different heat-treatment conditions and to reveal the regulations on its corrosion residual strength for the prediction of the alloy’s service life. Given GW63K alloy’s significant potential for shipbuilding applications, 3.5 wt.% NaCl solution was selected to evaluate the corrosion performance under simulated seawater conditions.

## 2. Materials and Methods

### 2.1. Sample Preparation

The material used in this work is semi-continuous casting GW63K (Mg-6.07Gd-2.89Y-0.78Zr, wt.%) alloy, which was provided by the Shanghai spaceflight precision machinery institute, Shanghai China. The chemical composition was verified using inductively coupled plasma optical emission spectroscopy (ICP-OES 5900, Agilent, Santa Clara CA, USA). Specimens in as-cast, solution heat treatment (T4), and ageing (T6) conditions were prepared by wire cutting machine and grounded on Al_2_O_3_ waterproof abrasive paper. After the specimens were polished until the scratches were removed, they were cleaned by ethanol and dried in air. The T4 treatment includes annealing at 525 °C for 8 h and quenching to room temperature. The T6 heat treatment includes a T4 treatment before aging at 200 °C in an air furnace for 80 h to achieve the peak-aged state. Argon gas shielding was employed during the heat treatment process to prevent oxidation.

### 2.2. Immersion Test

The 3.5 wt.% NaCl solution was made with analytic reagent chemicals and distilled water. The pH value was adjusted to 7.0. The corrosion experiments were carried out at the room temperature of 25 °C. The immersion corrosion specimens were prepared with standardized dimensions of 15 × 15 × 15 mm³, and an average mass of 6.20 ± 0.02 g. Specimens were immersed into the corrosion medium simultaneously and taken out after 1, 2, 4, 8, 24, 48, and 72 h. The immersed specimens were cleaned by aqueous solutions of 1 wt.% AgNO_3_ and 10 wt.% CrO_3_ to fully erase the corroded products before rinsing in water and ethanol and drying in air. The specimens were weighed before and after immersion. Specimens masses were measured pre- and post-corrosion using an analytical balance with a precision of ±0.0001 g.

### 2.3. Tensile Sample and Tensile Test

**Figure 1:**
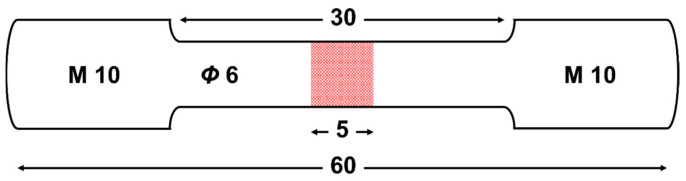
Schematic of specimen for tensile test. The center area (in red) is for pre-corrosion when needed.

Tensile tests were conducted using a UTM 5105 electronic universal testing machine (Suns, Shenzhen China). GW63K alloy specimens under different heat treatment conditions were machined according to the dimensions shown in Figure 1. A number of the tensile specimens were subjected to pre-corrosion on the center surface regions (red area in Figure 1) prior to the tensile test. Specimens were partly immersed into 3.5 wt.% NaCl aqueous solutions for 24, 48, and 72 h at the room temperature of 25 °C. Localized corrosion was achieved by a silicone rubber coating to designated specimen surfaces. Post-immersion, the protective layer was completely removed through a single incision without surface residue or mechanical damage. The tensile specimen was cleaned as described in Section 2.2 for the immersion specimen.

### 2.4. Analysis

Microstructure was examined by an optical microscope (OM, Axio Imager A2m, Carl Zeiss, Oberkochen, Germany), a field-emission scanning electron microscope (SEM, Auriga Field Emission Scanning Electron Microscope, Carl Zeiss, Oberkochen, Germany), and a field-emission transmission electronic microscope. Specimens were etched in 3% nitric acid solution for 10s prior to OM and SEM observation. Depth of the pits was determined with a three-dimensional confocal laser scanning microscope (CLSM, OLS 4000 LEXT, Olympus, Tokyo, Japan).

## 3. Results

### 3.1. Immersion Test Corrosion

**Figure 2:**
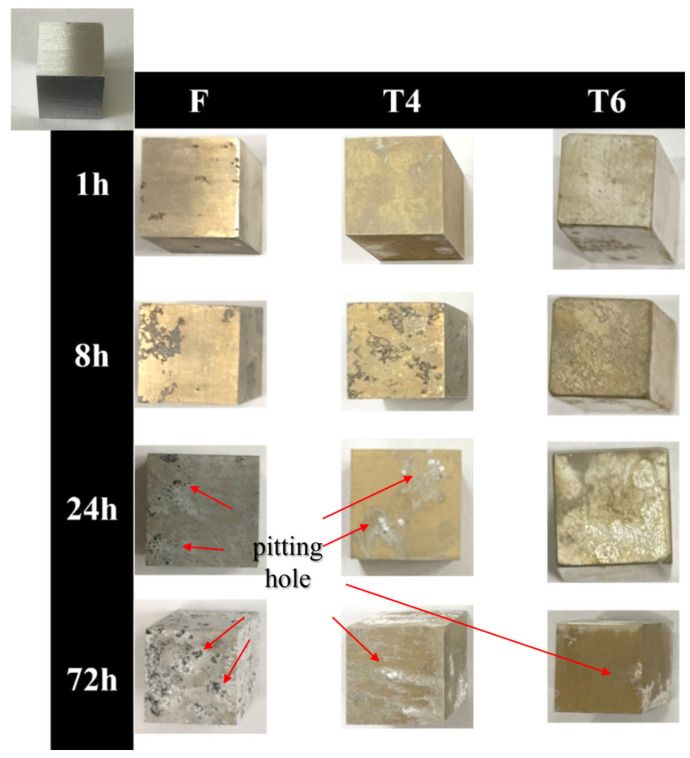
Specimens before and after the immersion test.

The GW63K alloy specimens under different heat treatment conditions exhibited distinct corrosion surface evolution with immersion time (Figure 2). During the initial corrosion stage, pitting holes occurred across the specimen surfaces. As corrosion progressed, both pit growth and new pit formation were observed. It is revealed that T4- and T6-treated specimens demonstrated significantly reduced pit density compared to as-cast specimens.

**Figure 3:**
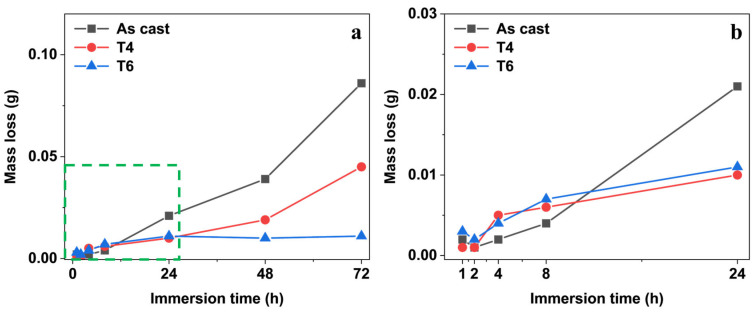
Corrosion mass loss of GW63K specimens in 3.5 wt.% NaCl solution within immersion time (**a**), and the enlarged plot within the green box for the first 24 h (**b**).

Figure 3 indicates the mass change of GW63K Mg alloy specimens as a function of corrosion time. With prolonged immersion time, the mass of specimens under different heat treatment conditions exhibited a continuous decline. Among all conditions, the as-cast specimens demonstrated the highest mass loss (approximately 0.10 g after 72 h), which was nearly double that of the T4-treated specimens. The T6-treated specimens showed negligible mass change during the 72-h test period. The findings indicate that the heat treatment significantly mitigated the corrosion-induced mass loss in the specimen, as both solution treatment (T4) and artificial aging treatment (T6) enhance the corrosion resistance of GW63K Mg alloy.

### 3.2. Tensile Test

**Figure 4:**
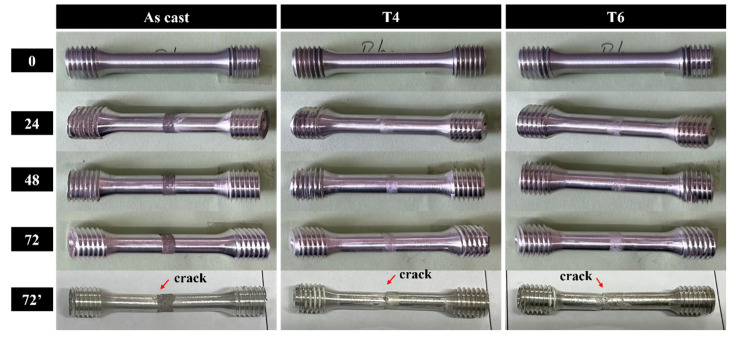
Tensile test specimens. (72′ indicates specimens that have undergone both 72-h immersion and subsequent tensile testing).

**Figure 5:**
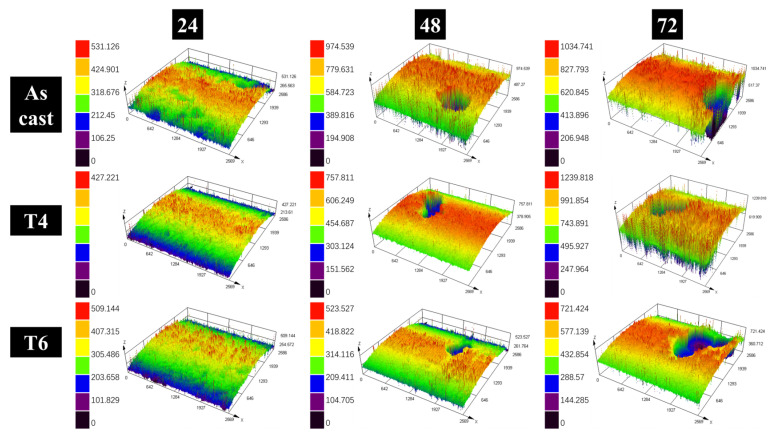
Surface of pre-corrosion zone of tensile test specimens.

In this study, GW63K magnesium alloy tensile specimens were subjected to controlled pre-corrosion treatment prior to tensile testing. A well-defined corrosion zone was intentionally introduced on each specimen, with subsequent tensile fractures consistently originating within these pre-corroded regions (Figure 4). Quantitative characterization of corroded surface morphology was performed using 3D confocal laser scanning microscopy, with results presented in Figure 5. Analysis of Figure 4 and Figure 5 yields the following conclusions, consistent with the experimental findings in Section 3.1: 1. Corrosion severity exhibits a time-dependent escalation, showing significant intensification with prolonged immersion duration. 2. The corrosion resistance followed the hierarchy: T6 > T4 > as-cast.

Figure 5 further demonstrates the progressive enlargement of corrosion pits with extended immersion time. Notably, substantial pit growth became evident after 48 h of exposure, while T6-treated specimens maintained significantly smaller pit dimensions throughout the testing period.

**Figure 6:**
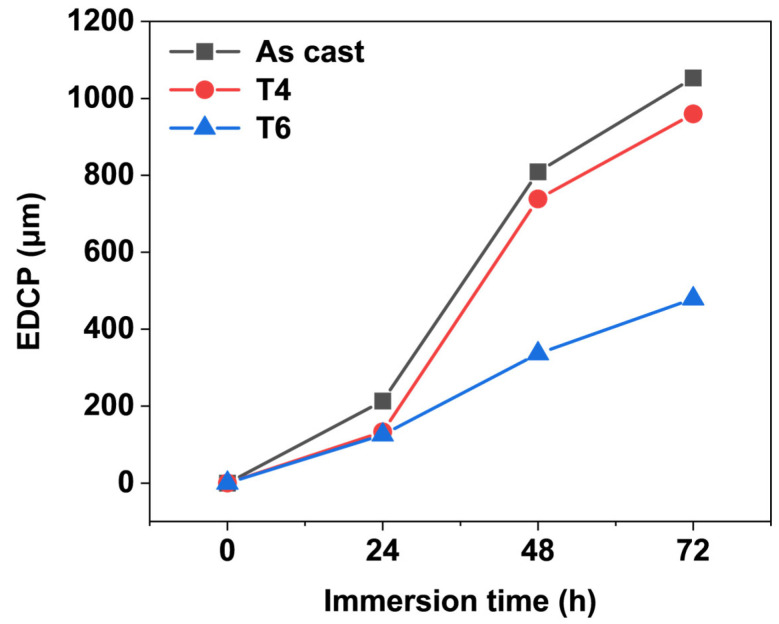
Extreme depth of corrosion pits on tensile test specimens.

The maximum values of the depth of corrosion pits on each specimen were recorded as the extreme depth of the corrosion pits (EDCP), as illustrated in Figure 6. The EDCP of specimens under different heat treatment conditions exhibited a progressive increase with corrosion time up to 72 h. After 72 h of immersion, the as-cast specimen displayed the highest EDCP value (~1 mm), whereas the T6-treated specimen showed a significantly reduced EDCP (479 μm). These results demonstrate that the growth of corrosion pits in the heat-treated alloy is effectively suppressed. The finding aligns with the immersion test results presented in Section 3.1, confirm that the corrosion resistance of GW63K Mg alloy is markedly enhanced by heat treatment.

**Figure 7:**
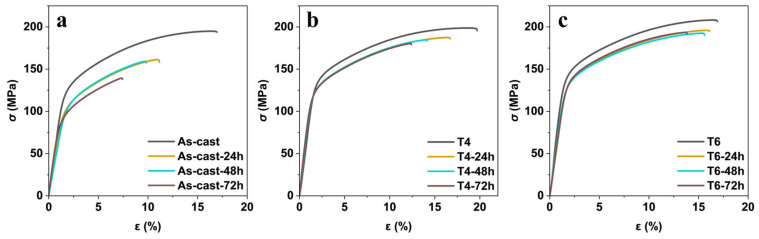
Stress-strain curves of GW63K Mg alloy in (**a**) as-cast, (**b**) T4, and (**c**) T6 conditions.

**Figure 8:**
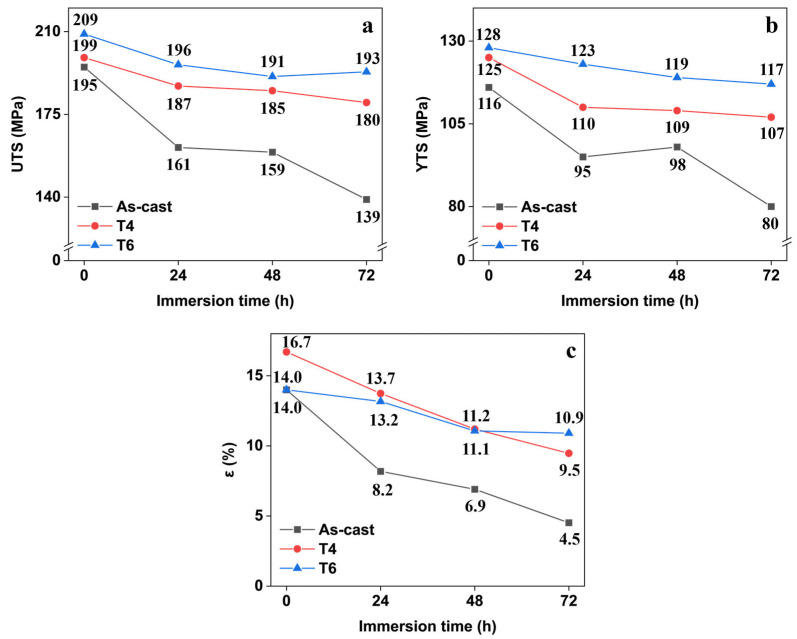
The variation of (**a**) ultimate tensile strength, (**b**) yield tensile strength, and (**c**) fracture elongation with immersion time.

Figure 7 exhibits the stress-strain curve of the GW63K alloy in as-cast, T4, and T6 conditions after pre-corrosion treatment for 0, 24, 48, and 72 h in 3.5 wt.% NaCl aqueous solution. These tensile curves exhibited nearly identical characteristics: the slope of the elastic deformation stage remained unchanged, followed by distinct plastic deformation until fracture occurred. This result indicates that the fracture mode of GW63K alloy under different heat treatment conditions with varied immersion times was not affected by corrosion induced degradation within 72 h. However, a pronounced trend was observed where the strength of the specimens significantly dropped with prolonged corrosion time. The ultimate tensile strength, yield tensile strength, and the fracture elongation versus immersion time are summarized in Figure 8.

As illustrated in Figure 8, all specimens exhibited progressive reductions in ultimate tensile strength, yield strength, and elongation with increasing corrosion time within 72 h, regardless of heat treatment conditions. The as-cast specimen demonstrated the most severe degradation, with its tensile strength decreasing by 28.7% (from 195 MPa to 139 MPa), yield strength declining 31.0% (116 MPa to 80 MPa), and elongation plummeting 67.9% (14.0% to 4.5%). In contrast, T4- and T6-treated specimens showed superior corrosion resistance. For instance, the T6 specimen retained 92.3% of its original tensile strength (a mere 16 MPa reduction) after 72 h of corrosion, while maintaining elongation above 10%. These results demonstrate that corrosion significantly compromises the tensile properties of GW63K magnesium alloy. Furthermore, heat treatment effectively mitigates corrosion-induced mechanical degradation by enhancing the alloy’s inherent corrosion resistance.

## 4. Discussion

The variation of mass loss, EDCP, and strength with the corrosion time indicates that the studied magnesium alloy in NaCl solution presents an accelerating corrosion with time. Corrosion results in degradation of corrosion residual strength, post-corrosion yield strength, and fracture elongation. Pitting corrosion serves as the primary factor responsible for the degradation of post-corrosion tensile properties of the GW63K alloy. This might be attributed to the reduction in the real load-bearing cross-section caused by corrosion pits, which induces stress concentration and deteriorates intergranular bonding. On the other hand, the corrosion resistance of GW63K magnesium alloy was significantly improved after heat treatment. Microstructural evolution is the key factor responsible for enhanced corrosion resistance. Therefore, the variation of the microstructure, the effect of growing corrosion pits, and strength variation with the corrosion kinetics are discussed for understanding the strength degradation mechanism.

### 4.1. Microstructure

The corrosion resistance of GW63K Mg alloy is strongly associated with the microstructure.

**Figure 9:**
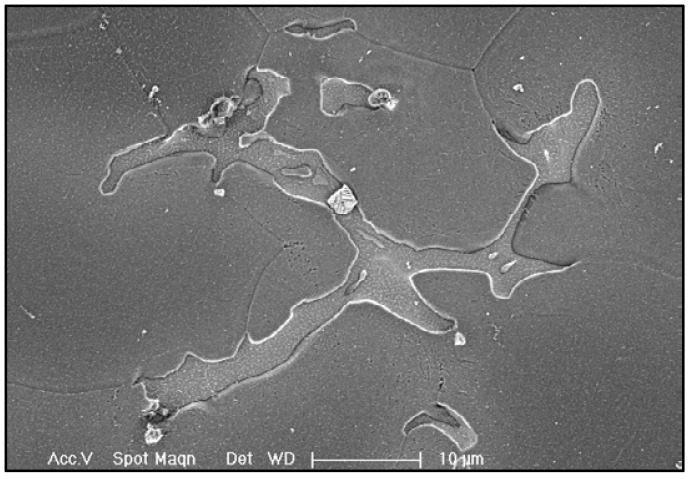
Mg_24_(Gd, Y)_5_ in GW63K Mg alloy.

**Figure 10:**
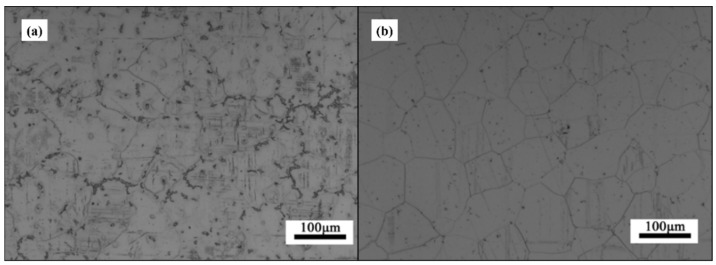
OM graph of (**a**) as-cast and (**b**) T4-GW63K Mg alloy.

Evidence shows that the as-cast GW63K Mg alloy is rich in eutectic phase Mg_24_(Gd, Y)_5_, which is mainly distributed near grain boundaries, as in Figure 9. The eutectic phase has higher electric potential than the Mg matrix, thus generating a corrosion microcell with the Mg matrix nearby when corrosion happens [6,7,14]. The Mg matrix, as the anode, keeps losing electrons during the corrosion process and dissolves into the corroded solution, which is aqueous NaCl in this test. After heat solution treatment, the eutectic phases dissolve, as in Figure 10b, so the amount of corrosion microcells in the Mg alloy is significantly reduced, thus preventing the initiation of corrosion. Eventually, the corrosion resistance of heat solution treated GW63K Mg alloy is enhanced compared to as-cast alloy.

**Figure 11:**
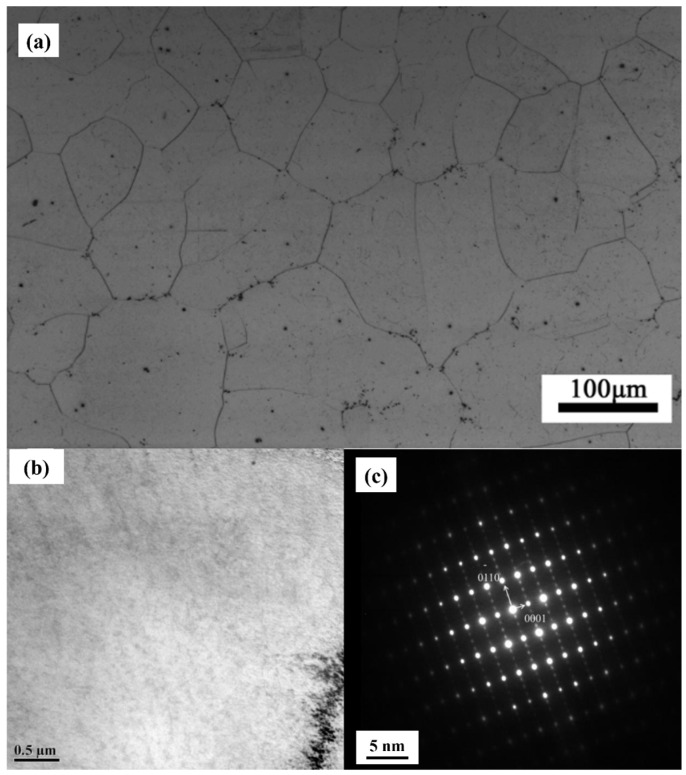
(**a**) OM, (**b**) SEM, and (**c**) TEM of aging precipitates of T6-GW63K Mg alloy.

Aging precipitations should be responsible for further improvement of corrosion resistance of GW63K-T6 alloy [20]. After aging treatment, nanoscale aging precipitates occurred in the grain of GW63K-T6 alloy, as in Figure 11, which performs as a barrier that inhibits the propagation of corrosion cracks. Hence, the corrosion resistance of alloy is enhanced.

### 4.2. Corrosion Pits

Corrosion significantly affected the tensile behavior of GW63K alloy. Pitting is the main characteristic of Mg and its alloys’ corrosion. The presence of corrosion pits results in a reduction in the cross-sectional area of the tensile specimens that leads to an increase of actual load [17]. Additionally, pitting always leads to stress concentration at the bottom of the corrosion pits [15,16]. Moreover, corrosion pits shall grow to grain boundaries as the corrosion continues and affect the bonding between magnesium grains nearby, thus lowering the intergranular adhesive force [21].

**Figure 12:**
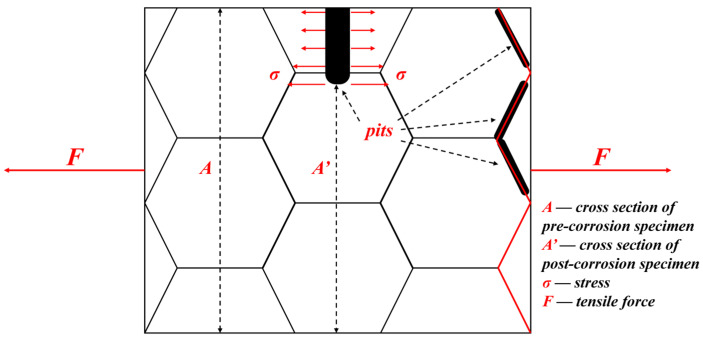
Schematic of corrosion pits affecting tensile fracture.

A schematic includes the above-mentioned aspects explaining how the tensile strength of corroded GW63K alloy is reduced (Figure 12). Therefore, as the time of pre-corrosion increases, both the amount and the size of corrosion pits grow, which leads to further reduction of tensile strength (Figure 5).

After heat treatment, the corrosion resistance of GW63K alloy is enhanced so that the amount and the scale of corrosion pits were both reduced. Therefore, the effect of corrosion on the tensile properties of the alloy are restrained than in the as-cast condition.

### 4.3. Corrosion Residual Strength

The corrosion residual strength (post-corrosion tensile strength) critically determines the load-bearing limits of engineering structures in corrosive environments and serves as a key lifespan predictor. Thus, assessing the corrosion residual strength of magnesium alloys is essential for evaluating their durability in corrosive service conditions.

**Figure 13:**
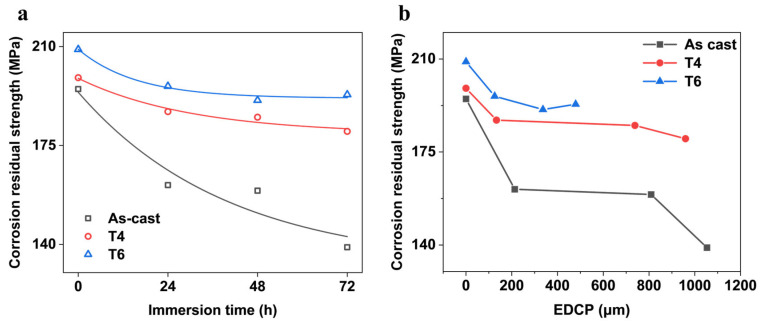
The variation of corrosion residual strength of GW63K alloy versus (**a**) immersion time and (**b**) extreme depth of corrosion pits within 72 h.

Figure 13a reflects the variation of corrosion residual strength of GW63K Mg alloy over immersion time within 72 h. It is indicated that the residual strength decreases rapidly in the initial stage of corrosion and eventually stabilizes as corrosion progresses. Such variation trend is similar to the attenuation of corrosion residual strength during the corrosion process in pitting-dominated magnesium alloys, such as AZ91 magnesium alloy [15,16,17]. As reported, the corrosion residual strength of exhibits a negative exponential dependence on corrosion time, which can be expressed as follows:

(1) $$\sigma_{CRS}=\sigma_{CV}+\sigma_{\lambda }\exp\left(\frac{-t}{A}\right)$$

where “*σ*_CRS_” represents the corrosion residual strength (MPa), “*σ*_CV_” is the critical value, which can be considered as the critical working strength (MPa); “*σ_λ_*” is the amplitude of attenuation caused by corrosion (MPa); “*t*” is the corrosion time (h); and “*A*” is the time constant. *σ*_CV_ + *σ*_λ_ = *σ*_0_, where “*σ*_0_” is the original tensile strength of the sample.

In the present study, according to Equation (1) and the results in Figure 13a, the critical working strengths of GW63K alloy under different heat treatment conditions are 132, 179, and 192 MPa, respectively. The exceptionally high critical working strength demonstrated by T6 alloy confirms significant improvement in allowable service stress in corrosive environments (45% increase vs. as-cast condition) and resistance to corrosion-induced fractures and failures.

The variation of corrosion residual strength of GW63K alloy with the dimensions of the extreme depth of the corrosion pits within 72 h is exhibited in Figure 13b. The corrosion residual strength decreases with increasing extreme depth of corrosion pits. The trend resembles that of AZ91 alloy [15,16,17], albeit with subtle differences. For AZ91 alloy, pitting corrosion serves as the dominant factor leading to strength degradation, where the corrosion residual strength of the alloy exhibits a negative linear correlation with the dimension of the extreme depth of corrosion pits. These findings imply that factors beyond pitting corrosion contribute to the residual strength degradation in GW63K magnesium alloy. For instance, RE elements in the alloy may inhibit the growth of corrosion cracks. To fully explain the mechanism of the reduction of the corrosion residual strength of GW63K alloy, further investigation is required.

## 5. Conclusions

The effect of corrosion on tensile behavior of GW63K (Mg-6Gd-3Y-Zr) magnesium alloy under different heat treatment conditions was studied in this paper. The conclusions can be expressed as follows:

1. Pitting corrosion serves as the primary factor responsible for the degradation of post-corrosion tensile properties of the GW63K alloy. Corrosion results in degradation of corrosion residual strength, post-corrosion yield strength, and fracture elongation.

2. The corrosion residual strength of GW63K alloy is closely dependent on the dimension of the extreme depth of the corrosion pits within 72 h. With prolonged corrosion time, the extreme depth of corrosion pits increases, leading to a corresponding decrease in corrosion residual strength.

3. The corrosion resistance of GW63K magnesium alloy was significantly improved after heat treatment. The corrosion resistance follows the declining order of T6 > T4 > as-cast. The aging precipitates in T6 alloy enhance corrosion resistance. The tensile properties of the T6 GW63K magnesium alloy after corrosion were also improved correspondingly. The corrosion residual strength, post-corrosion yield strength and the fracture elongation were observed to be increased.

## Data Availability

The original contributions presented in this study are included in the article. Further inquiries can be directed to the corresponding author.
